# Distinguishing clinical efficacy from vagal target engagement in perioperative transcutaneous auricular vagus nerve stimulation

**DOI:** 10.1080/07853890.2026.2749299

**Published:** 2026-10-16

**Authors:** Yi Zhang

**Affiliations:** Department of Anesthesiology, Chongqing Wanzhou Health Center for Women and Children, Chongqing, People’s Republic of China

Dear Editor

Yuan and colleagues report a randomized, sham-controlled trial in which perioperative transcutaneous auricular vagus nerve stimulation (taVNS) was associated with lower cough pain and reduced postoperative opioid use after thoracoscopic partial lung resection [1]. The clinical signal is of interest, but two details of the stimulation protocol make the physiological interpretation less certain.

First, stimulation intensity was individualized by increasing current until the participant reported clear tingling or pricking and then reducing it to a subthreshold level. That procedure is understandable for the preoperative session. The second session, however, was delivered after skin incision during general anesthesia. The report does not explain whether the preoperative setting was reused, whether a fixed current was selected, or whether another rule was applied. The actual delivered current is also not reported for either group. Consensus reporting standards for transcutaneous vagus nerve stimulation recommend reporting both how stimulation dose is selected and the dose actually applied [2]. Without that information, the intraoperative session cannot be reproduced exactly with respect to stimulation intensity, although the stimulation site, frequency, pulse width, duty cycle, timing, and duration are reported.

Second, the sham condition was electrical stimulation of the earlobe rather than an inactive control. Earlobe stimulation is often used as a sham, but it should not automatically be assumed to be physiologically inert. Cheng et al. found changes in heart rate and heart rate variability during earlobe stimulation [3]. A study already cited by Yuan et al. also showed that transcutaneous greater auricular nerve stimulation through earlobe electrodes altered brainstem connectivity; both the earlobe-stimulation and taVNS groups showed reductions in pain, although no untreated group was included [4]. These findings do not establish that earlobe stimulation is analgesic in the perioperative setting. They do show that the comparator itself may be biologically active.

The potentially active nature of earlobe stimulation does not invalidate the randomized between-group comparison. Rather, the trial estimates the effect of cymba conchae stimulation relative to electrically active earlobe stimulation. It therefore does not establish efficacy relative to an inert control or, by itself, isolate a specifically vagal mechanism. Clarification from Yuan and colleagues regarding how stimulation intensity was determined during general anesthesia, including whether the preoperative setting was reused, would improve both the reproducibility and interpretation of the trial.

Sincerely,

Yi Zhang*Department of Anesthesiology, Chongqing Wanzhou Health Center for Women and Children, Chongqing, People’s Republic of China*joyfairy00@outlook.com


## Data Availability

No new data were generated or analysed for this correspondence.

## Supplementary Material

Manuscript Highlighted Revised.docx
